# Comparison of the efficacy and safety of ultrasound-guided surgery with traditional surgery for plasma cell mastitis: a systematic review and meta-analysis

**DOI:** 10.3389/fmed.2025.1596231

**Published:** 2025-07-11

**Authors:** Ti Zhang, Chang Yao, Jianzhong Shi, Shikun Ma, Yue Hu

**Affiliations:** ^1^Department of Breast Surgery, Yangzhou Hospital of TCM, Yangzhou Jiangsu, China; ^2^Department of Breast Surgery, Jiangsu Province Hospital of Chinese Medicine, Nanjing Jiangsu, China

**Keywords:** plasma cell mastitis, ultrasound-guided surgery, traditional surgery, efficacy, safety

## Abstract

**Objectives:**

This study aims to offer an updated, comprehensive comparison of the efficacy and safety between ultrasound-guided surgery (UGS) and traditional surgery (TS) for plasma cell mastitis (PCM).

**Methods:**

Studies comparing UGS with conventional surgery for PCM were retrieved from Embase, PubMed, Web of Science, China National Knowledge Infrastructure (CNKI), and Wanfang databases (up to March 2025). Sensitivity and subgroup analyses were conducted to assess result stability and identify sources of heterogeneity. All findings were evaluated using Grading of Recommendations Assessment, Development and Evaluation (GRADE). Analyses were performed with Review Manager 5.4 and STATA 15.0.

**Results:**

Nine eligible studies, including 951 patients (486 UGS, 465 TS), were analyzed. Baseline characteristics were comparable between the groups. Pooled analysis indicated that UGS resulted in shorter operative time, lower postoperative complication rates, reduced intraoperative hemorrhage, lower visual analog scale (VAS) scores, improved efficacy, and higher satisfaction with overall appearance. Recurrence rates and pain satisfaction were comparable between the two groups. According to GRADE classification, results for efficacy, satisfaction, and mammary deformity were rated as low-quality evidence, while other outcomes were rated as very low-quality due to significant heterogeneity, imprecision, or publication bias.

**Conclusion:**

Evidence suggests that UGS improves efficacy, satisfaction, and reduces postoperative complications for PCM. However, given the limited quality and quantity of the included studies, further high-quality research is needed to confirm these findings.

**Systematic review registration:**

https://www.crd.york.ac.uk/PROSPERO/.

## 1 Introduction

Plasma cell mastitis (PCM) is a chronic, recurrent inflammatory breast lesion, a form of nonlactating mastitis, commonly observed in nonlactating women aged 30–50 years and occasionally in men. PCM represents 1.4 to 5.4% of benign breast diseases, with an increasing incidence in recent years (1). The pathogenesis and etiology of PCM remain unclear, although some studies suggest associations with congenital breast tissue abnormalities, lactogen imbalance, smoking, and psychotropic drugs. Clinically, PCM presents as breast lumps, pain, and abscesses, typically divided into overflow, lump, pus, and rupture phases. These phases often overlap, complicating treatment and making PCM a challenging clinical condition.

Current clinical treatment primarily targets disease manifestations, using methods such as abscess incision and drainage. However, surgical excision and drainage significantly affect breast appearance and have high recurrence rates, failing to meet aesthetic expectations. Ultrasound has recently proven beneficial for early PCM diagnosis (2), and ultrasound-guided interventions, combined with various methods (e.g., medications, traditional Chinese medicine), have emerged as promising treatments. These approaches offer ease of operation, accurate positioning, minimal trauma, reduced pain, and satisfactory post-healing breast appearance.

Comparative analyses of the efficacy, safety, and recurrence rates of ultrasound-guided puncture and drainage versus conventional surgical excision have not been reported. We present a pooled analysis and updated evidence comparing the efficacy, safety, postoperative complication rates, and recurrence rates of ultrasound-guided surgery (UGS) and traditional surgery (TS) for PCM treatment.

## 2 Materials and methods

### 2.1 Data search

This evidence-based analysis followed the PRISMA 2020 Statement and was registered with International Prospective Register of Systematic Reviews (PROSPERO) (Registered ID: CRD42023416273). We systematically searched literature published up to March 2025 in China National Knowledge Infrastructure (CNKI), Wanfang, Embase, PubMed, and Web of Science to compare the efficacy and safety of UGS versus TS for PCM treatment. We used the following terms: “Ultrasonography,” “Ultrasound,” “Diagnostic Ultrasound,” “Diagnostic Ultrasounds,” “Ultrasound, Diagnostic,” “Ultrasounds, Diagnostic,” “Ultrasound Imaging,” “surgery,” “plasma cell mastitis,” and “mammary duct ectasia.” See Supplementary Table S1 for the search strategy. We manually reviewed the reference lists of relevant studies. Two researchers independently retrieved and evaluated the studies, with disputes resolved by consensus.

### 2.2 Determination of applicable studies

Studies meeting the following criteria were included: (1) randomized controlled or cohort studies; (2) adult subjects with PCM or mammary duct ectasia; (3) comparisons of ultrasound-guided surgery and traditional surgery; (4) at least one perioperative outcome assessed (e.g., intraoperative hemorrhage, operating time, healing time, hospital stay, appearance satisfaction, pain satisfaction, visual analog scale (VAS) score, local infection, postoperative subcutaneous stasis, mammary deformity, scar length, postoperative complication rate, recurrence rate, and efficacy); and (5) sufficient data to calculate the odds ratio (OR) or weighted mean difference (WMD). We excluded syntheses, letters, case reports, editorial comments, pediatric studies, conference summaries, and unpublished studies. Ductal carcinoma, defined as a precursor lesion of PCM, was included.

### 2.3 Data collection

Data collection was independently performed by two researchers, with disagreements resolved by a third. We extracted data on study time, first author, publication date, research country, study design, sample size, age, follow-up duration, disease course, operating time, intraoperative hemorrhage, healing time, hospital stay, appearance satisfaction, pain satisfaction, VAS score, local infection, postoperative subcutaneous stasis, mammary deformity, scar length, postoperative complication rate, recurrence rate, and efficacy. When continuous variables were presented as medians, we calculated mean ± standard deviation using established methods (3, 4). Missing or undisclosed data were obtained by contacting the authors.

### 2.4 Quality evaluation

Study quality was assessed using the Newcastle-Ottawa Scale (NOS), with scores of 7–9 indicating high quality. The Cochrane Handbook for Systematic Reviews of Interventions 5.1.0 was used to assess randomized controlled trial (RCT) quality based on seven criteria: random sequence generation, allocation concealment, participant and staff blinding, outcome assessment blinding, incomplete result data, selective reporting, and other bias sources (5). Studies were rated as low, high, or unclear risk. Low-risk bias evaluations were considered superior. Both researchers independently assessed study quality and resolved divergences through discussion.

### 2.5 Data analysis

A random-effects model was used when significant heterogeneity was present; otherwise, a fixed-effects model was applied. One-way sensitivity analyses assessed the impact of included studies on outcomes with significant heterogeneity. Funnel plots were generated in Review Manager version 5.4 (Cochrane Collaboration, Oxford, UK), and Egger’s regression test (6) in Stata version 15.1 (Stata Corp, College Station, TX, USA) was used to assess publication bias, with *p* < 0.05 indicating significant bias. Grading of recommendations assessment, development and evaluation (GRADE) was used to evaluate and grade the evidence for each outcome as “high,” “moderate,” “low,” or “very low” quality. We conducted subgroup analyses of recurrence rates, efficacy rates, and GRADE classifications, categorizing the interventions into paracentesis and nonparacentesis groups. The paracentesis group included: ultrasound-guided paracentesis or rrigation, ultrasound-guided inimally invasive rotational resection, ultrasound-guided microwave ablation. The nonparacentesis group comprised: abscess incision and drainage, mass excision.

## 3 Results

### 3.1 Literature search and study characteristics

**Figure 1:**
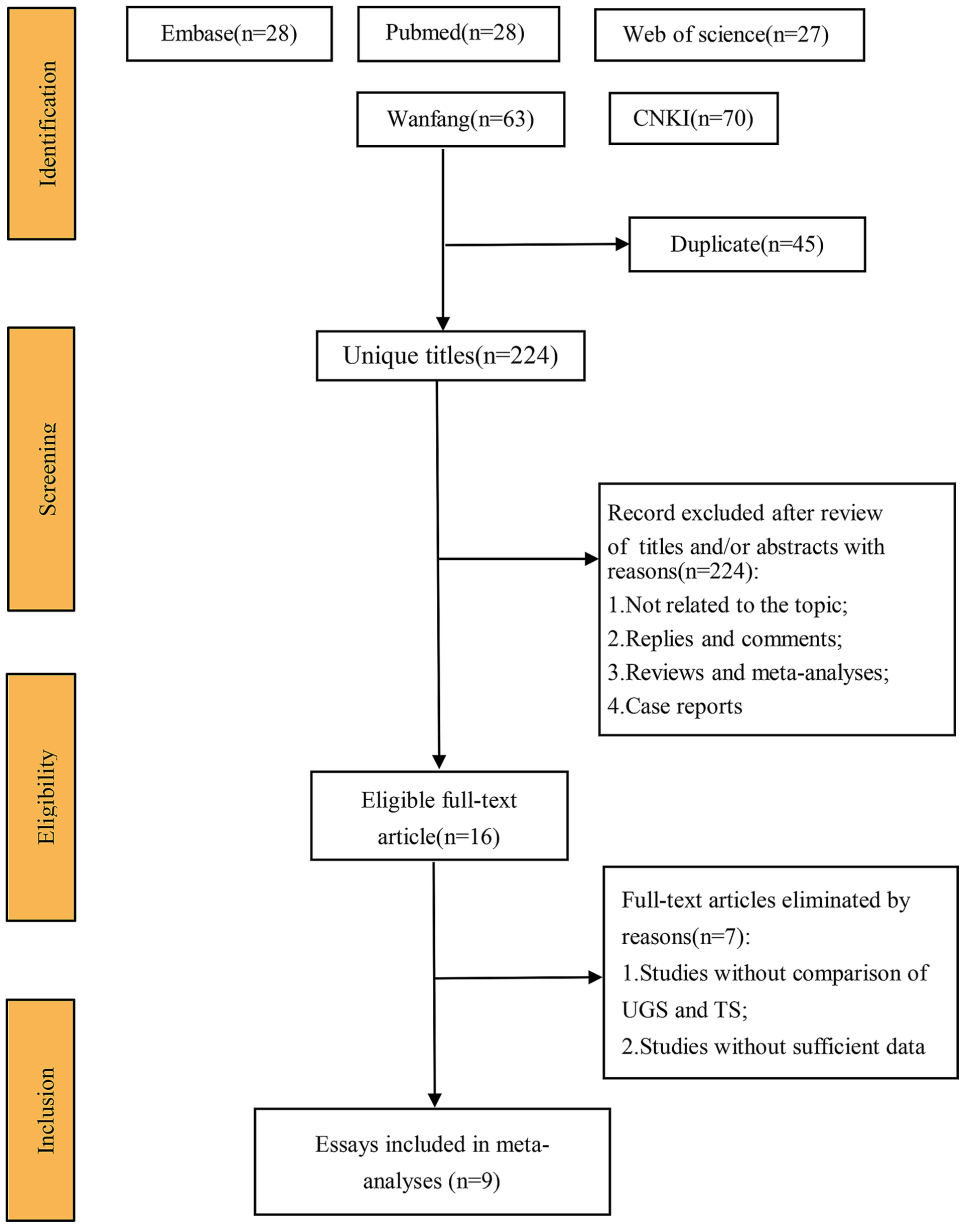
Flow sheet of the systematic search and selection course.

Figure 1 illustrates the search and selection flow diagram. A systematic search identified 269 studies from Embase (*n* = 28), PubMed (*n* = 81), Web of Science (*n* = 27), CNKI (*n* = 70), and Wanfang (*n* = 63).

After removing duplicates, 224 titles and abstracts were screened, resulting in 9 full-text articles with 951 cases (486 UGS vs. 465 TS) for pooled analysis (7–15). These comprised 5 prospective cohort studies (7, 8, 11, 12, 14) and 4 prospective randomized studies (9, 10, 13, 15). Table 1 presents the characteristics, evidence evaluation, and quality scores of the included studies. Supplementary Table S2 provides details of the quality assessment.

**Table 1:** Baseline features of applicable research and methodological evaluation.

Authors	Study period	Type of surgery	Study design	Patients (*n*)	Median follow-up (months)
		UGS/TS		UGS/TS	
H. L Wu et al.	2011–2013	Ultrasound-guided microwave ablation/ lesion resection	prospective	38/30	12
Jia et al.	2013–2016	Ultrasound-guided paracentesis/ segment resection	prospective	89/101	12
Luo et al.	2017–2018	Ultrasound-guided paracentesis/ segment resection	RCT	25/25	12
Zhou et al.	2013–2016	Ultrasound-guided paracentesis / Surgical incision and drainage	RCT	42/41	12
Yu et al.	2017–2019	EnCor minimally invasive surgery/ open surgery	RCT	60/60	6
Song et al.	2018–2020	EnCor minimally invasive surgery/ open surgery	prospective	43/43	6
Fu et al.	2019–2020	ultrasound-guided small-incision minimally invasive rotational resection / traditional surgery	RCT	30/30	3
Zhu et al.	2017	Ultrasound-guided paracentesis/ Surgical incision and drainage	prospective	40/40	12
Zhou et al.	2017–2019	ultrasound-guided microwave ablation/ traditional surgery	prospective	119/95	6–36

### 3.2 Demographic traits

No significant differences were found in mass diameter (WMD: 0.02; 95% confidence interval (CI): −0.14, 0.14; *p* = 0.98), location (WMD: 0.97; 95% CI: 0.69, 1.37; *p* = 0.86), single lesion (WMD: 0.80; 95% CI: 0.45, 1.44; *p* = 0.46), or crater nipple (WMD: 1.05; 95% CI: 0.51, 2.16; *p* = 0.90) between groups. Significant heterogeneity was observed in age (*p* < 0.00001; *I*^2^ = 85%) and disease course (*p* < 0.00001; *I*^2^ = 92%) (Table 2).

**Table 2:** Demographics data and clinical features of applicable research.

Outcomes	Studies	No. of patients	WMD or OR	95% CI	*p*-value	Heterogeneity			
		UGS/TS				Chi^2^	df	*p*-value	*I*^2^ (%)
Age (years)	8	367/370	0.87	[−1.22, 2.96]	0.41	47.66	7	<0.00001	85
Mass diameter	3	115/114	0.02	[−0.14, 0.14]	0.98	0.28	2	0.87	0
Location	5	255/259	0.97	[0.69, 1.37]	0.86	1.48	4	0.83	0
Single lesion	2	66/71	0.80	[0.45, 1.44]	0.46	0.27	1	0.60	0
Crater nipple	2	80/71	1.05	[0.51, 2.16]	0.90	0.17	1	0.68	0
Course of disease	3	144/156	−1.76	[−5.99,2.46]	0.41	25.33	2	<0.00001	92

OR, odds ratio; CI, confidence interval.; WMD, weighted mean difference.

### 3.3 Operating time

**Figure 2:**
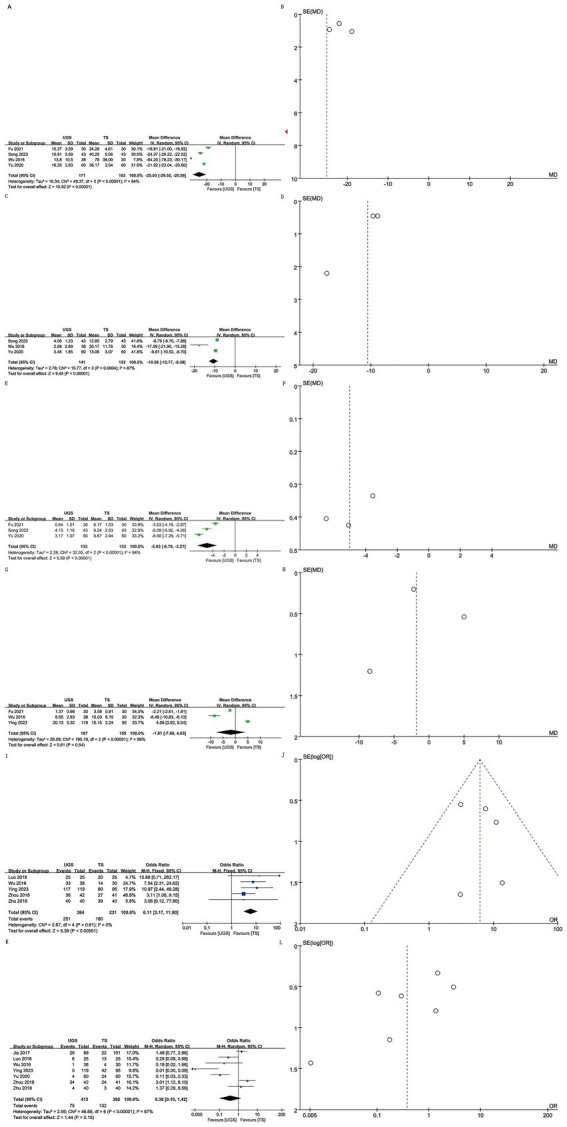
The forest and funnel plots. **(A,B)** Operating time; **(C,D)** intraoperative hemorrhage; **(E,F)** Healing time; **(G,H)** Hospital stays; **(I,J)** Efficacy; **(K,L)** Recurrence.

Data on operating time were extracted from 4 studies involving 334 patients (171 UGS vs. 163 TS) (10–12, 15). Pooled analysis showed significantly shorter operative times in the UGS group (WMD: -25.05; 95% CI: −29.55, −20.56; *p* < 0.00001), with notable heterogeneity (*I*^2^ = 94%, *p* < 0.00001) (Figure 2A). The funnel plot suggested minimal publication bias (Figure 2B), although Egger’s test showed no statistical significance (*p* = 0.303).

### 3.4 Intraoperative hemorrhage

Analysis of intraoperative hemorrhage included 3 studies with 274 patients (141 UGS vs. 133 TS) (10–12). Pooled analysis showed significantly lower hemorrhage in the UGS group (WMD: -10.58; 95% CI: −12.77, −8.38; *p* = 0.0004), with notable heterogeneity (*I*^2^ = 87%, *p* < 0.00001) (Figure 2C). Egger’s test showed no significant publication bias (*p* = 0.207) (Figure 2D).

### 3.5 Healing time

Three studies with 266 patients (133 UGS vs. 133 TS) were analyzed (10, 12, 15). Data showed significantly shorter healing time in the UGS group (WMD: −0.53; 95% CI: −6.79, −3.27; *p* < 0.00001) (Figure 2E), with substantial heterogeneity (*I*^2^ = 94%, *p* < 0.00001). Egger’s test showed no significant publication bias (*p* = 0.401) (Figure 2F).

### 3.6 Hospital stays

Three articles reported hospital stay data for 342 patients (187 UGS vs. 155 TS) (8, 11, 15). No significant differences were found (WMD: –1.81; 95% CI: −7.66, 4.03; *p* = 0.54) (Figure 2G), with no apparent heterogeneity or publication bias (*I*^2^ = 99%, *p* < 0.00001; Egger’s test, *p* = 0.877) (Figure 2H).

### 3.7 Efficacy

Data on efficacy included 495 patients (264 UGS vs. 231 TS) from five studies (7–9, 11, 13). Pooled analysis showed higher efficacy in the UGS group (OR: 6.11; 95% CI: 3.17, 11.8; *p* < 0.00001) (Figure 2I), with no heterogeneity (*I*^2^ = 0%, *p* = 0.61) or publication bias (Egger’s test, *p* = 0.641) (Figure 2J).

### 3.8 Recurrence

Seven articles reported recurrence data for 805 patients (413 UGS vs. 392 TS) (7–11, 13, 14). Analysis showed similar efficacy in both groups (OR: 0.38; 95% CI: 0.10, 1.42; *p* = 0.15) (Figure 2K), with significant heterogeneity (*I*^2^ = 87%, *p* < 0.00001). No publication bias was detected (Egger’s test, *p* = 0.112; Figure 2L).

### 3.9 Appearance satisfaction

**Figure 3:**
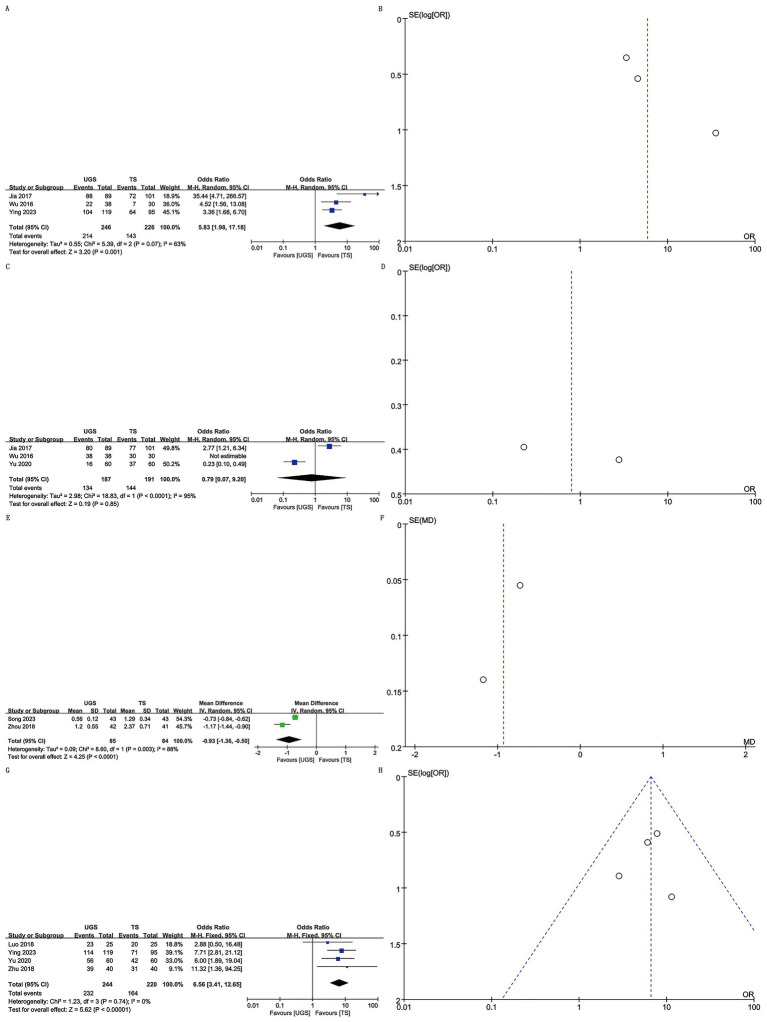
The forest and funnel plots. **(A,B)** Appearance satisfaction; **(C,D)** Pain satisfaction; **(E,F)** VAS score; **(G,H)** Satisfaction.

Three studies involving 472 patients (246 UGS vs. 226 TS) were analyzed (8, 11, 14). Pooled analysis showed significantly higher appearance satisfaction in the UGS group (OR: 5.83; 95% CI: 1.98, 17.18; *p* = 0.001) (Figure 3A) and notable heterogeneity (*I*^2^ = 63%, *p* = 0.07). No publication bias was found, either statistically (Egger’s test, *p* = 0.163) or visually (Figure 3B).

### 3.10 Pain satisfaction

Three studies with 378 patients (187 UGS vs. 191 TS) reported on pain satisfaction (10, 11, 14). No significant differences were found between groups (OR: 0.79; 95% CI: 0.07, 9.20; *p* = 0.85) (Figure 3C), despite substantial heterogeneity (*I*^2^ = 95%, *p* < 0.00001) (Figure 3D).

### 3.11 VAS score

Two studies reported VAS score data for 169 patients (85 UGS vs. 84 TS) (9, 12). Analysis revealed a significant decrease in VAS score in the UGS group (WMD: –0.93; 95% CI: −1.36, −0.50; *p* < 0.0001) with substantial heterogeneity (*I*^2^ = 88%, *p* = 0.003) (Figure 3E). No publication bias was detected (Figure 3F).

### 3.12 Satisfaction

Four studies involving 464 patients (244 UGS vs. 220 TS) reported satisfaction data (7, 8, 10, 13). Combined evidence showed higher satisfaction in the UGS group (OR: 6.56; 95% CI: 3.41, 12.65; *p* < 0.00001) (Figure 3G) with no heterogeneity (*I*^2^ = 0%, *p* = 0.74) or publication bias (Egger’s test, *p* = 0.812) (Figure 3H).

### 3.13 Local infection

**Figure 4:**
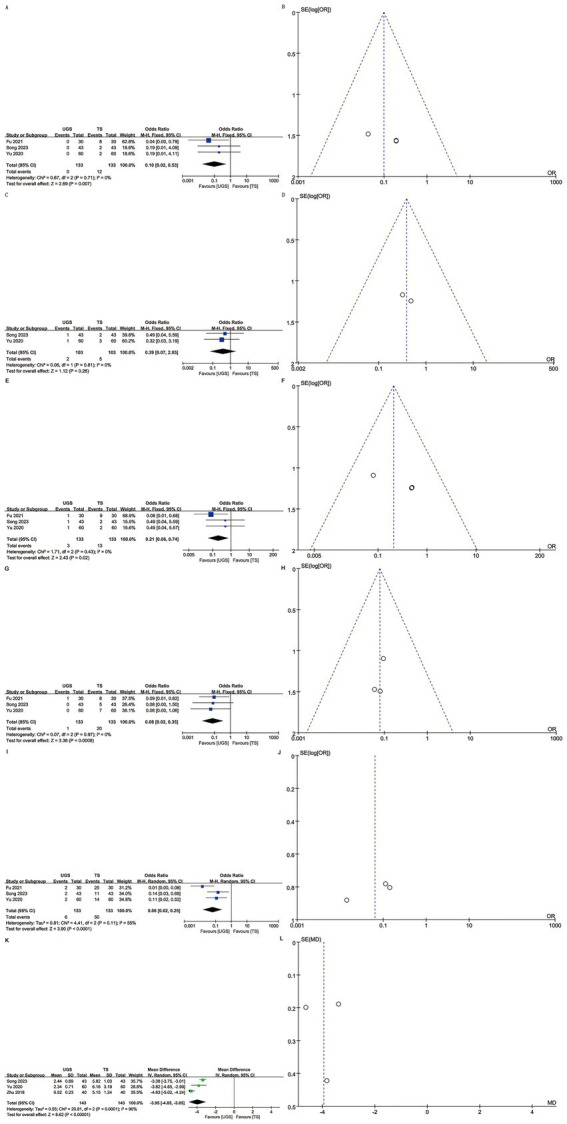
The forest and funnel plots. **(A,B)** Local infection; **(C,D)** Skin ecchymosis; **(E,F)** Subcutaneous stasis; **(G,H)** Mammary deformity; **(I,J)** Total complications; **(K,L)** Scar length.

Three articles reported local infection rates in 266 patients (133 UGS vs. 133 TS) (10, 12, 15). Analysis showed a lower infection rate in the UGS group (OR: 0.10; 95% CI: 0.02, 0.53; *p* = 0.007) (Figure 4A) with no significant heterogeneity (*I*^2^ = 0%, *p* = 0.71) or visual bias (Figure 4B). Egger’s test showed slight publication bias (*p* = 0.033).

### 3.14 Skin ecchymosis

Two articles involving 206 patients (103 UGS vs. 103 TS) reported skin ecchymosis (10, 12). Postoperative data showed no significant difference (OR: 0.39; 95% CI: 0.07, 2.05; *p* = 0.26) (Figure 4C) with no significant heterogeneity (*I*^2^ = 0%, *p* = 0.81) or visual bias (Figure 4D).

### 3.15 Subcutaneous stasis

Three articles analyzed subcutaneous stasis in 266 patients (133 UGS vs. 133 TS) (10, 12, 15). Pooled results showed a significantly lower risk of subcutaneous stasis (OR: 0.21; 95% CI: 0.06, 0.74; *p* = 0.02) in the UGS group (Figure 4E) with no notable heterogeneity (*I*^2^ = 0%, *p* = 0.43) (Figure 4F). Egger’s test indicated slight publication bias (*p* = 0.021).

### 3.16 Mammary deformity

Data on mammary deformity were obtained from three studies involving 266 patients (133 UGS vs. 133 TS) (10, 12, 15). Results showed a significantly lower risk of mammary deformity in the UGS group (OR: 0.08; 95% CI: 0.02, 0.35; *p* = 0.0008) (Figure 4G). No evidence of publication bias or significant heterogeneity (*I*^2^ = 0%, *p* = 0.97) was found, either statistically (Egger’s test, *p* = 0.413) or visually (Figure 4H).

### 3.17 Total complications

Three articles addressed total complications in 266 patients (133 UGS vs. 133 TS) (10, 12, 15). Pooled results showed a significantly lower risk of total complications (OR: 0.06; 95% CI: 0.02, 0.25; *p* < 0.0001) in the UGS group (Figure 4I). Heterogeneity (*p* = 0.11, *I*^2^ = 55%) and publication bias, both visual (Figure 4J) and statistical (Egger’s test, *p* = 0.211), were not significant.

### 3.18 Scar length

Three studies assessed scar length in 286 patients (143 UGS vs. 143 TS) (7, 10, 12). Results showed a significant reduction in scar length in the UGS group (WMD: –3.95; 95% CI: −4.85, −3.05; *p* < 0.00001). Heterogeneity was significant (*p* < 0.0001, *I*^2^ = 90%) (Figure 4K). No publication bias was detected, both statistically (Egger’s test, *p* = 0.999) and visually (Figure 4L).

### 3.19 Sensitivity analysis

We performed a one-way sensitivity test for intraoperative bleeding, operative time, hospital stay, healing time, recurrence rate, pain satisfaction, total complications, and cosmetic satisfaction. The effect of each study on the WMD was assessed by sequentially excluding individual studies. Sensitivity tests showed that the combined WMD remained unchanged after excluding any individual studies on intraoperative bleeding (Supplementary Figure S1A), operative time (Supplementary Figure S1B), hospital stay (Supplementary Figure S1C), healing time (Supplementary Figure S1D), recurrence rate (Supplementary Figure S1E), pain satisfaction (Supplementary Figure S1F), total complications (Supplementary Figure S1G), and cosmetic satisfaction (Supplementary Figure S1H). Excluding Fu et al.’s 2021 study (15) removed heterogeneity in total complications (*I*^2^ = 0%, *p* = 0.84), indicating its significant contribution to heterogeneity. Similarly, excluding Jia et al.’s 2017 study (14) removed heterogeneity in cosmetic satisfaction (*I*^2^ = 0%, *p* = 0.65), suggesting its major role in heterogeneity. Excluding Wu et al.’s 2016 study (11) reduced heterogeneity in intraoperative bleeding (*p* = 0.21, *I*^2^ = 36%), indicating significant heterogeneity from this study.

### 3.20 Subgroup analysis of recurrence

According to the different intervention measures, recurrence was divided into ultrasound-guided paracentesis and nonparacentesis groups. The paracentesis group included 4 studies involving 403 patients (196 UGS versus 207 TS) (7, 9, 13, 14). No significant difference was found between the two groups (OR: 1.21; 95% CI: 0.50, 2.98; *p* = 0.67), but heterogeneity was significant (I^2^ = 66%, *p* = 0.03). The nonparacentesis group comprised 3 studies with 402 patients (217 UGS versus 185 TS) (8, 10, 11). Data showed a significant difference (OR: 0.06; 95% CI: 0.01, 0.42; *p* = 0.005) and high heterogeneity (*I*^2^ = 66%, *p* = 0.06) (Supplementary Figures S2A,B).

Subgroup analysis based on research methods classified recurrence into cohort study and RCT groups. The cohort group included 4 studies with 552 patients (286 UGS versus 266 TS) (7, 8, 11, 14). The RCT group included 3 studies with 253 patients (127 UGS versus 126 TS) (9, 10, 13). Evidence synthesis showed no significant difference in either group (cohort: WMD: 0.26; 95% CI: 0.02, 3.11; *p* = 0.29; RCT: WMD: 0.46; 95% CI: 0.06, 3.57; *p* = 0.46), but higher heterogeneity (cohort: *I*^2^ = 89%, *p* < 0.00001; RCT: *I*^2^ = 90%, *p* < 0.00001) (Supplementary Figures S3A,B).

### 3.21 Subgroup assessment of efficacy

Efficacy was assessed in two groups: ultrasound-guided paracentesis and nonparacentesis, based on intervention measures. Three studies in the paracentesis group included 213 patients (107 UGS versus 106 TS) (7, 9, 13). Pooled analysis showed higher efficacy in the paracentesis group than in the nonparacentesis group (OR: 3.98; 95% CI: 1.55, 10.24; *p* = 0.004). Heterogeneity was insignificant (*I*^2^ = 0%, *p* = 0.64). Two studies in the nonparacentesis group included 282 patients (157 UGS versus 125 TS) (8, 11). The data indicated a significant difference between the two groups (OR: 8.98; 95% CI: 3.53, 22.84; *p* < 0.00001). Heterogeneity was minimal (*I*^2^ = 0%, *p* = 0.70) (Supplementary Figures S4A,B).

### 3.22 GRADE rating of outcomes

In the GRADE classification, all results except efficacy, satisfaction, and mammary deformity were rated as very low-quality evidence due to significant heterogeneity, imprecision, or publication bias. These three outcomes were rated as low-quality evidence (Table 3).

**Table 3:** GRADE rating of outcomes.

Outcomes	Risk of bias	Inconsistency	Indirectness	Imprecision	Publication bias	Plausible confounding	Magnitude of effect	Dose–response gradient	GRADE
Operating time	No serious risk	Serious inconsistency	No serious indirectness	No serious imprecision	Undetected	Would not reduce effect	No	No	Very low
Intraoperative hemorrhage	No serious risk	Serious inconsistency	No serious indirectness	No serious imprecision	Undetected	Would not reduce effect	No	No	Very low
Healing time	No serious risk	Serious inconsistency	No serious indirectness	No serious imprecision	Undetected	Would not reduce effect	No	No	Very low
Hospital stays	No serious risk	Serious inconsistency	No serious indirectness	Serious imprecision	Undetected	Would not reduce effect	No	No	Very low
Efficacy	No serious risk	No serious inconsistency	No serious indirectness	No serious imprecision	Undetected	Would not reduce effect	No	No	Low
Recurrence	No serious risk	Serious inconsistency	No serious indirectness	Serious imprecision	Undetected	Would not reduce effect	No	No	Very low
Appearance satisfaction	No serious risk	Serious inconsistency	No serious indirectness	No serious imprecision	Undetected	Would not reduce effect	No	No	Very low
Pain satisfaction	No serious risk	Serious inconsistency	No serious indirectness	Serious imprecision	Undetected	Would not reduce effect	No	No	Very low
VAS score	No serious risk	Serious inconsistency	No serious indirectness	No serious imprecision	Undetected	Would not reduce effect	No	No	Very low
Satisfaction	No serious risk	No serious inconsistency	No serious indirectness	No serious imprecision	Undetected	Would not reduce effect	No	No	Low
Local infection	No serious risk	No serious inconsistency	No serious indirectness	Serious imprecision	Strongly suspected	Would not reduce effect	No	No	Very low
Skin ecchymosis	No serious risk	No serious inconsistency	No serious indirectness	Serious imprecision	Undetected	Would not reduce effect	No	No	Very low
Subcutaneous stasis	No serious risk	No serious inconsistency	No serious indirectness	Serious imprecision	Strongly suspected	Would not reduce effect	No	No	Very low
Mammary deformity	No serious risk	No serious inconsistency	No serious indirectness	No serious imprecision	Undetected	Would not reduce effect	No	No	Low
Total complications	No serious risk	Serious inconsistency	No serious indirectness	No serious imprecision	Undetected	Would not reduce effect	No	No	Very low
Scar length	No serious risk	Serious inconsistency	No serious indirectness	No serious imprecision	Undetected	Would not reduce effect	No	No	Very low

## 4 Discussion

Medical therapy for PCM presents significant challenges for subgroups. The primary treatment strategies include conservative and surgical approaches. Conservative treatments involve antibiotics, anti-tuberculosis drugs, immunosuppressants, hormonotherapy, and Chinese herbal medicine (8, 16, 17). Studies indicate that antibiotics are often ineffective against PCM (18, 19). Anti-tuberculosis drugs and Chinese herbal medicine require prolonged treatment with slow efficacy (20). Hormone treatment, though rapidly effective, carries a high risk of recurrence, with post-withdrawal recurrence often exceeding the primary lesion’s area (21).

Most surgeons currently prefer surgical resection for treating PCM (22). Surgical strategies include mass excision, incision and dilation, and incision and drainage (11, 23). Incision and drainage are not advised during the abscess stage due to the risk of delayed sinus tract formation. Advances in surgical techniques now enable patients with stage I abscesses to undergo segmentectomy and suture (8). Postoperative suture scars may affect the breast’s appearance and cause physical and psychological trauma (24). PCM treated with incision drainage alone has a recurrence rate of up to 79%, whereas segmental mastectomy reduces the recurrence rate to 28% (25). Segmental mastectomy has notable drawbacks, including extensive damage, prolonged postoperative dressing changes, partial loss of glandular function, and impact on breast aesthetics, making it less acceptable for many female patients (26). Based on our meta-analysis, UGS appears particularly suitable for younger patients with high aesthetic expectations due to several key advantages. First, it offers superior cosmetic results, including significantly reduced scar length (WMD: −3.95 cm), a lower incidence of breast deformity (OR: 0.08), and better preservation of the natural breast contour. Second, UGS is especially beneficial for reproductive-age women (typically 25–40 years), individuals with body image concerns, and those planning future pregnancy or lactation. Third, it provides notable psychosocial benefits, such as higher satisfaction with breast appearance (OR: 5.83), reduced psychological distress related to surgical scars, and improved quality of life indicators.

Ultrasound-guided minimally invasive surgery is widely used to treat PCM, achieving good therapeutic effects (8, 11). Its superiority over traditional surgery (including open surgery such as segmental resection and incision and drainage) in perioperative outcomes and postoperative recovery remains inconclusive. This meta-analysis assessed the efficacy and safety of ultrasound-guided versus traditional surgery for PCM. Results showed that ultrasound-guided minimally invasive surgery outperformed traditional surgery in perioperative outcomes, including operative time, intraoperative hemorrhage, healing time, scar length, VAS score, and risk of local infection. The advantages may stem from ultrasound’s ability to accurately locate lesions without incising subcutaneous tissue, thus shortening operative time and reducing mammary gland damage. The small incision, absence of internal sutures, and reduced intraoperative hemorrhage contribute to rapid postoperative recovery. The small wound minimizes the need for repeated dressing changes, significantly reducing the risk of complications such as infection and areola hematoma (11, 16).

Our study found similar recurrence rates for ultrasound-guided minimally invasive and traditional surgery for PCM. Subgroup analysis revealed that ultrasound-guided minimally invasive nonparacentesis surgery had a significantly lower recurrence rate, likely due to the higher lesion resection rate of the minimally invasive approach (27). This advantage was absent in the ultrasound-guided paracentesis subgroup, suggesting that ultrasound guidance may be more beneficial for nonparacentesis procedures, such as microwave ablation and minimally invasive rotational resection. Our study also revealed that PCM patients undergoing ultrasound-guided minimally invasive surgery had a lower risk of breast deformity and higher satisfaction. This likely results from direct lesion removal under ultrasound guidance, minimizing damage to surrounding breast tissue, preserving nipple and areola sensory function, and reducing skin pigmentation (28).

However, this meta-analysis has several limitations. First, the included studies had diverse surgical interventions, contributing to heterogeneity. Second, significant heterogeneity existed in some outcomes, and despite subgroup and sensitivity analyses, we could not identify potential sources. Third, most studies were from China, with insufficient data from other regions.

Despite these limitations, this is the first and largest meta-analysis evaluating the efficacy and safety of ultrasound-guided versus traditional surgery for PCM. The results confirm the superiority of ultrasound-guided surgery, as reported in previous studies. Current GRADE assessments classify the evidence as low to very low owing to several critical limitations. Most studies are single-center with small samples, and many are non-randomized. Marked heterogeneity stems from variations in surgical technique (e.g., aspiration versus microwave ablation) and follow-up duration, while reporting bias persists because long-term recurrence and quality-of-life outcomes are seldom documented. To strengthen the evidence base, future work should prioritize multicenter RCTs enrolling at least 200 participants and incorporating rigorous randomization and assessor blinding. Protocols must be standardized by defining ultrasound-guided surgical parameters, harmonizing follow-up intervals (12, 24, and 36 months), and adopting core outcome sets that include recurrence, complications, and patient satisfaction. Investigators should also follow consolidated standards of reporting trials (CONSORT) or strengthening the observational studies in epidemiology (STROBE) guidelines, report baseline characteristics and attrition comprehensively, and share datasets under findable, accessible, interoperable, reusable (FAIR) principles. This structured strategy will raise evidence quality and offer more reliable guidance for clinical practice.

## 5 Conclusion

This systematic review and meta-analysis indicate that UGS provides notable benefits in managing PCM. These include improved surgical efficiency, evidenced by shorter operative time, reduced intraoperative bleeding, and minimal surgical trauma. Postoperative recovery is enhanced through faster wound healing, fewer complications (e.g., infection, subcutaneous hematoma, breast deformity), and better quality of life. UGS also leads to greater cosmetic satisfaction, as its minimally invasive approach and concealed incisions improve breast appearance, making it especially suitable for younger women and patients with high aesthetic expectations. Subgroup analysis further suggests that non-aspiration techniques such as microwave ablation and minimally invasive rotational resection may reduce recurrence, offering valuable guidance for clinical decision-making. These results support the use of UGS as a first-line treatment for PCM, particularly in early-stage lesions or in patients seeking breast-conserving strategies.

Despite the proven benefits of UGS, current research presents notable limitations that require further investigation. Most existing studies are single-center with small sample sizes, underscoring the need for large-scale, multicenter RCTs to confirm the long-term efficacy and safety of UGS. Standardization of surgical protocols is essential, including clear indications, procedural guidelines, and comparative evaluation of various UGS techniques such as aspiration, microwave ablation, and minimally invasive resection to support evidence-based clinical decision-making. Follow-up durations are typically short (≤12 months), limiting assessment of recurrence, breast function, and psychological outcomes; longer-term data are needed. Further research into the molecular mechanisms of PCM, integrating imaging modalities (e.g., ultrasound elastography, MRI) and pathological biomarkers, could enable more precise disease classification and personalized treatment. Additionally, comparative cost-effectiveness analyses of UGS and traditional surgery would offer critical insights for healthcare resource allocation.

## Data Availability

The original contributions presented in the study are included in the article/Supplementary material, further inquiries can be directed to the corresponding author/s.
