# Supplementary material for: Comparison of the efficacy and safety of ultrasound-guided surgery with traditional surgery for plasma cell mastitis: a systematic review and meta-analysis
Source: Front Med (Lausanne). 2025 Jul 11;12:1596231. doi: 10.3389/fmed.2025.1596231 (PMC12289682; doi:10.3389/fmed.2025.1596231)
Supplement: Supplementary file 1 [file Data_Sheet_1.docx]

**Supplementary materials**

**Table S1.** Detailed search strategy in three databases.

| Database | Search strategy |
| --- | --- |
| PubMed | ((("Ultrasonography"[Mesh]) OR ((((((Ultrasound) OR (Diagnostic Ultrasound)) OR (Diagnostic Ultrasounds)) OR (Ultrasound, Diagnostic)) OR (Ultrasounds, Diagnostic)) OR (Ultrasound Imaging))) AND (surgery)) AND ((plasma cell mastitis) OR (mammary duct ectasia)) |
| Embase^*^ | 1 (Ultrasonography or Ultrasound or Diagnostic Ultrasound or Diagnostic Ultrasounds or Ultrasound, Diagnostic or Ultrasounds, Diagnostic or Ultrasound Imaging).af.  2 (surgery).af.  3 (plasma cell mastitis or mammary duct ectasia).af.  4 1 and 2 and 3 |
| Web of Science | 1 Ultrasonography (All Fields) or Ultrasound (All Fields) or Diagnostic Ultrasound (All Fields) or Diagnostic Ultrasounds (All Fields) or Ultrasound, Diagnostic (All Fields) or Ultrasounds, Diagnostic (All Fields) or Ultrasound Imaging (All Fields)  2 surgery (All Fields)  3 plasma cell mastitis (All Fields) or mammary duct ectasia (All Fields)  4 #3 AND #2 AND #1 |

**Notes:** ^*^ We retrieved articles from Embase via the Ovid (https://ovidsp.ovid.com/).

**Table S2.** Quality evaluation of the eligible studies with Newcastle–Ottawa scale

| Study | Selection | | | | Comparability | | Outcome | | |
| --- | --- | --- | --- | --- | --- | --- | --- | --- | --- |
|  | Representative-ness | Selection of  non-exposed | Ascertainment  of exposure | Outcome not present at start | Comparability on most important factors | Comparability on other risk factors | Assessment of outcome | Long enough follow-up (≥6months) | Adequacy  (completeness) of follow-up |
| Jia et al | * | * | * | * | * | - | * | * | * |
| Song et al | * | * | * | * | * | - | * | * | * |
| Wu et al. | * | * | * | * | * | - | * | * | * |
| Zhou et al. | * | * | * | * | * | - | * | * | * |
| Zhu et al. | * | * | * | * | * | - | * | * | * |

**Notes:** ^*^indicates criterion met; - indicates significant of criterion not met.

**
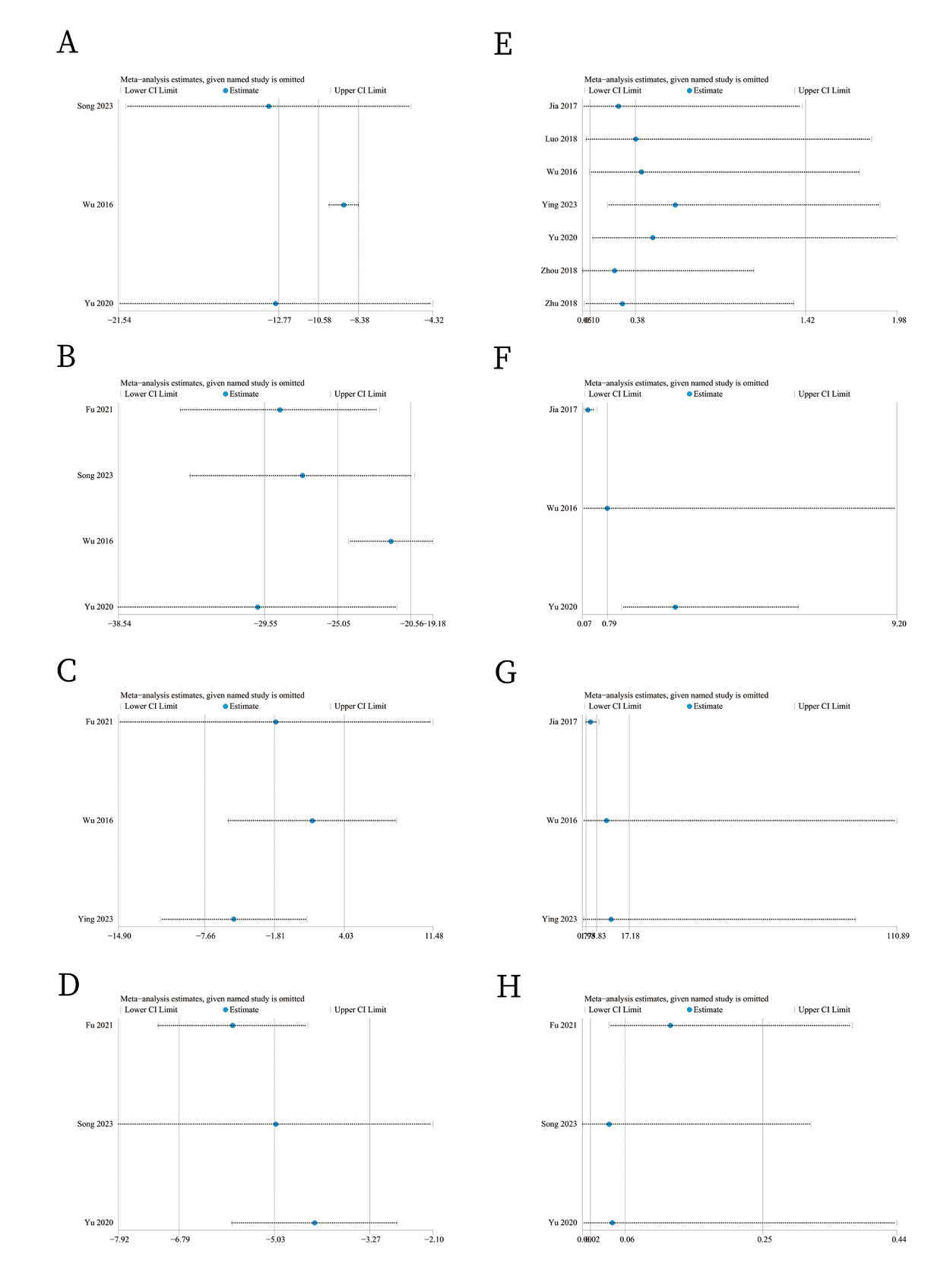
**

**Figure S1** Sensitivity analysis. **A** Intraoperative hemorrhage. **B** Time of surgery. **C** Hospital stays. **D** Healing time. **E** Recurrence. **F** Pain satisfaction. **G** Total complications. **H** Appearance satisfaction


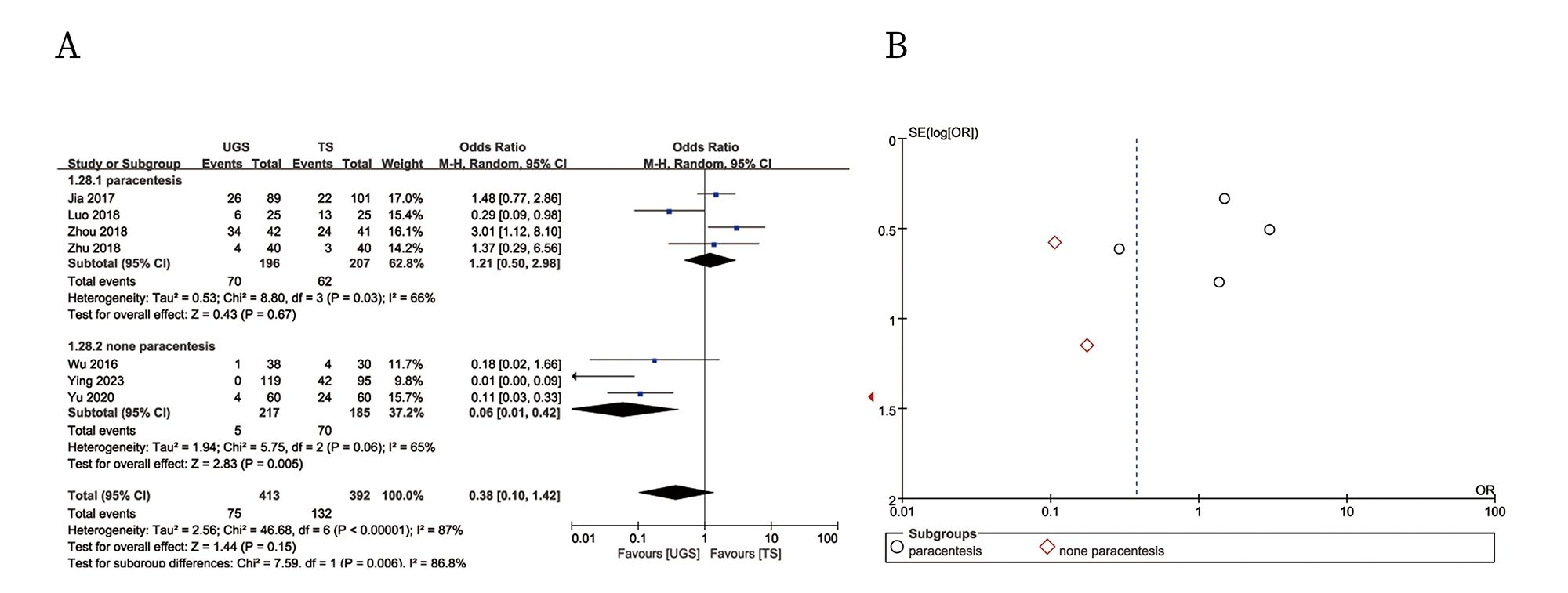


**Figure S2** **A** Forest plots of the recurrence subgroup analysis. **B** Funnel plots of the recurrence subgroup analysis


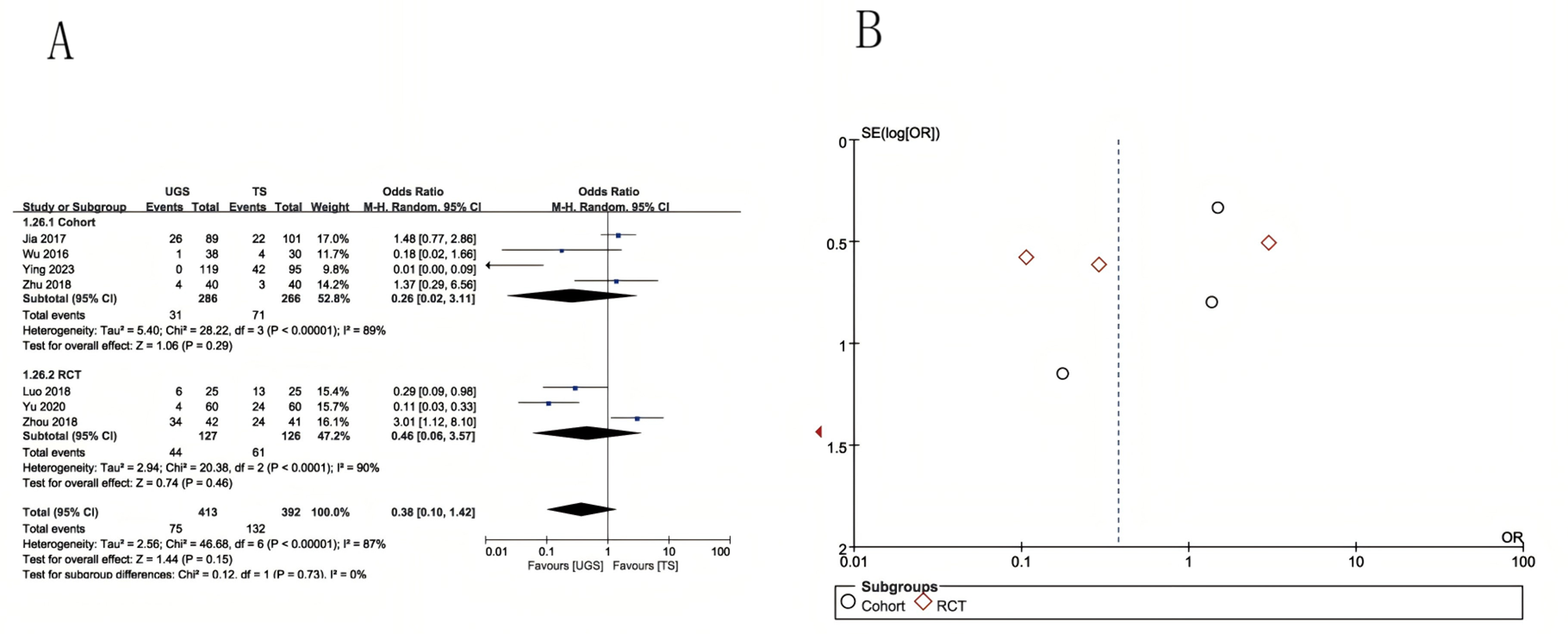


**Figure S3 A** Forest plots of the recurrence subgroup analysis: study type. **B** Funnel plots of the recurrence recurrence subgroup analysis: study type.

**
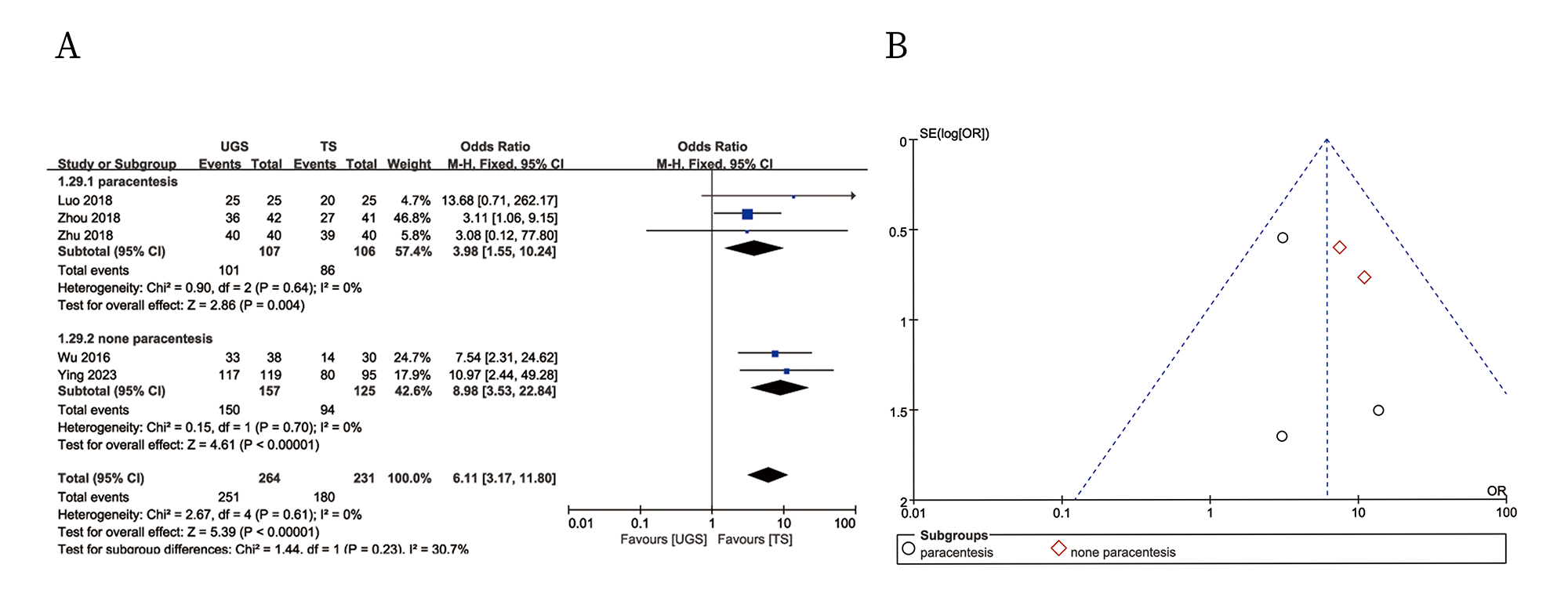
**

**Figure S4** **A** Forest plots of the efficacy subgroup analysis. **B** Funnel plots of the efficacy subgroup analysis
